# Effects of human limb gestures on galvanic coupling intra-body communication for advanced healthcare system

**DOI:** 10.1186/s12938-016-0192-z

**Published:** 2016-05-26

**Authors:** Xi Mei Chen, Sio Hang Pun, Jian Feng Zhao, Peng Un Mak, Bo Dong Liang, Mang I. Vai

**Affiliations:** State Key Laboratory of Analog and Mixed-Signal VLSI, University of Macau, Avenida da Universidade, Taipa, Macau, China; Electrical and Computer Engineering, University of Macau, Avenida da Universidade, Taipa, Macau, China; Shenzhen Polytechnic, West Shahe Street Xili Lake, Nanshan District, Shenzhen, 518000 Guangdong Province China

**Keywords:** Human limb gestures, Joint angle, Muscle fatigue, Bit error rate, Galvanic coupling intra-body communication

## Abstract

**Background:**

Intra-Body Communication (IBC), which utilizes the human body as the transmission medium to transmit signal, is a potential communication technique for the physiological data transfer among the sensors of remote healthcare monitoring system, in which the doctors are permitted to remotely access the healthcare data without interrupt to the patients’ daily activities.

**Methods:**

This work investigates the effects of human limb gestures including various joint angles, hand gripping force and loading on galvanic coupling IBC channel. The experiment results show that channel gain is significantly influenced by the joint angle (i.e. gain variation 1.09–11.70 dB, *p* < 0.014). The extension, as well as the appearance of joint in IBC channel increases the channel attenuation. While the other gestures and muscle fatigue have negligible effect (gain variation <0.77 dB, *p* > 0.793) on IBC channel. Moreover, the change of joint angle on human limb IBC channel causes significant variation in bit error rate (BER) performance.

**Conclusions:**

The results reveal the dynamic behavior of galvanic coupling IBC channel, and provide suggestions for practical IBC system design.

## Background

The aging population and prevalence of chronic diseases call for the deployment of remote health monitoring system [1, 2], which helps to improve the quality of patients’ life meanwhile prevents the deterioration of diseases. The healthcare monitoring data from the wearable sensors, which are limited in memory and energy, should be timely and reliably delivered to a relay node mounted on the body for further processing. The transmission technique utilized by the relay node and sensors, especially the implantable sensors such as pacemaker should be low transmission power (energy saving) [3], low interference (avoid interference from or to other telemetry devices) and high reliability (avoid information leakage from eavesdropping) [4]. Compared to the existing wireless techniques (i.e. Bluetooth, Wi-Fi) which are pervasively utilized in the everyday devices (e.g. cell phone, microwave even, earphone), galvanic coupling IBC is a potential candidate. As the signal in galvanic coupling IBC is confined within human body [5, 6], and thus it avoids signal leakage from eavesdropping and the interference to other devices. Moreover, it avoids interference from existing wireless techniques due to its low transmission frequency (i.e. <1 MHz [6, 7]). Compared to another type IBC- capacitive coupling IBC, where two ground electrodes at transceiver require floating [8], galvanic coupling IBC is less prone to environment interference due to its signal electrodes and ground electrodes at transceiver are attached on human body [6]. The floating electrodes in capacitive coupling IBC makes its implementation in implantable devices challenged. Generally, galvanic coupling IBC works in lower frequency than capacitive coupling IBC. The advantage of using low frequency carrier is minimizing the system clock [9], which can minimize the local heating and allow one to simplify the design of transceiver via low but at the expense of data rate. Fortunately, the data rate requirement for home-based healthcare data monitoring can be relatively low, e.g. 1.6 kbps in glucose monitor, 120 bps in body temperature surveillance and 144 kbps in ECG [10]. Therefore, the galvanic-type IBC serves as a promising choice to build the remote health monitoring system.

The remote health monitoring system permits the doctors or physicians to access the physiological data without interrupting the patients’ everyday life, in which human body executes the movement in terms of joint angle, force, torque, etc [11]. That is the human limb will move and post different gestures. However, the effects of different gestures on the communication performance of IBC channel have not been fully discussed yet. Few empirical measurements [12–15] have been conducted to investigate this issue. For instance, channel attenuation was found to be less influenced by the whole body motions such as sitting, standing and walking [16, 17]. The flexion of forearm caused channel gain in the upper limb capacitive coupling IBC channel vary around 2 dB [15]. It was found that with small transversal distance between electrodes, the performance of galvanic coupling method was more susceptible to the body composition while capacitive coupling method was affected by motion [13], the flexion of elbow joint (from downward to 90°) resulted in 5 dB attenuation decrease [14]. However, with large transversal distance between electrodes, wherein better attenuation results would be obtained for galvanic coupling method [16], the effects of different gestures are not yet investigated. It should be noted that the change of muscle condition such as isometric contraction (hand gripping force or loading) and muscle fatigue causes the change of electrical properties, i.e. electrical impedance of human arm decreases around 3–5 $\Omega$ due to isometric contraction [18], decreases 5–20 $\Omega$ [18, 19] owing to muscle fatigue [20], however, their effects on IBC channel have not yet been addressed. Moreover, the reliability of communication performance (i.e. BER) during dynamic behavior of human body is not discussed yet.

Consequently, in order to provide suggestions for galvanic coupling IBC system design, the effects on channel communication performance from different body gestures and muscle conditions are investigated. Although better channel gain was achieved in trunk and back for galvanic coupling method [16], however, the human limb channels will be investigated. Since the relay nodes, such as smart-watch or smart wristband [21, 22] are usually mounted on the human extremities. Specifically, the effects on human limb galvanic coupling IBC channels from different gestures, such as various joint angles, hand gripping force and hand loading, and muscle fatigue are yet to be studied. And the in-depth analysis of the effects on BER performance will be to conducted.

The signal propagation in galvanic coupling IBC channel can be characterized by the channel frequency response *H*(*f*) [23], which can be described as:

1 $$\begin{aligned} H(f) = \frac{V_{out}(f)}{V_{in}(f)}=|H(f)|e^{j\theta (f)} \end{aligned}$$

where *f* is operating frequency, $V_{out}(f)$ and $V_{in}(f)$ are the output signal and input signal, respectively. $\theta \, (f)$ is the phase, and |*H*(*f*)| is the magnitude of frequency response. In what follows, the experimental designs to measure the frequency response are presented. Then the experiment results and discussions are provided. Finally, the conclusions are provided.

## Methods

### Experiment setup

**Fig. 1:**
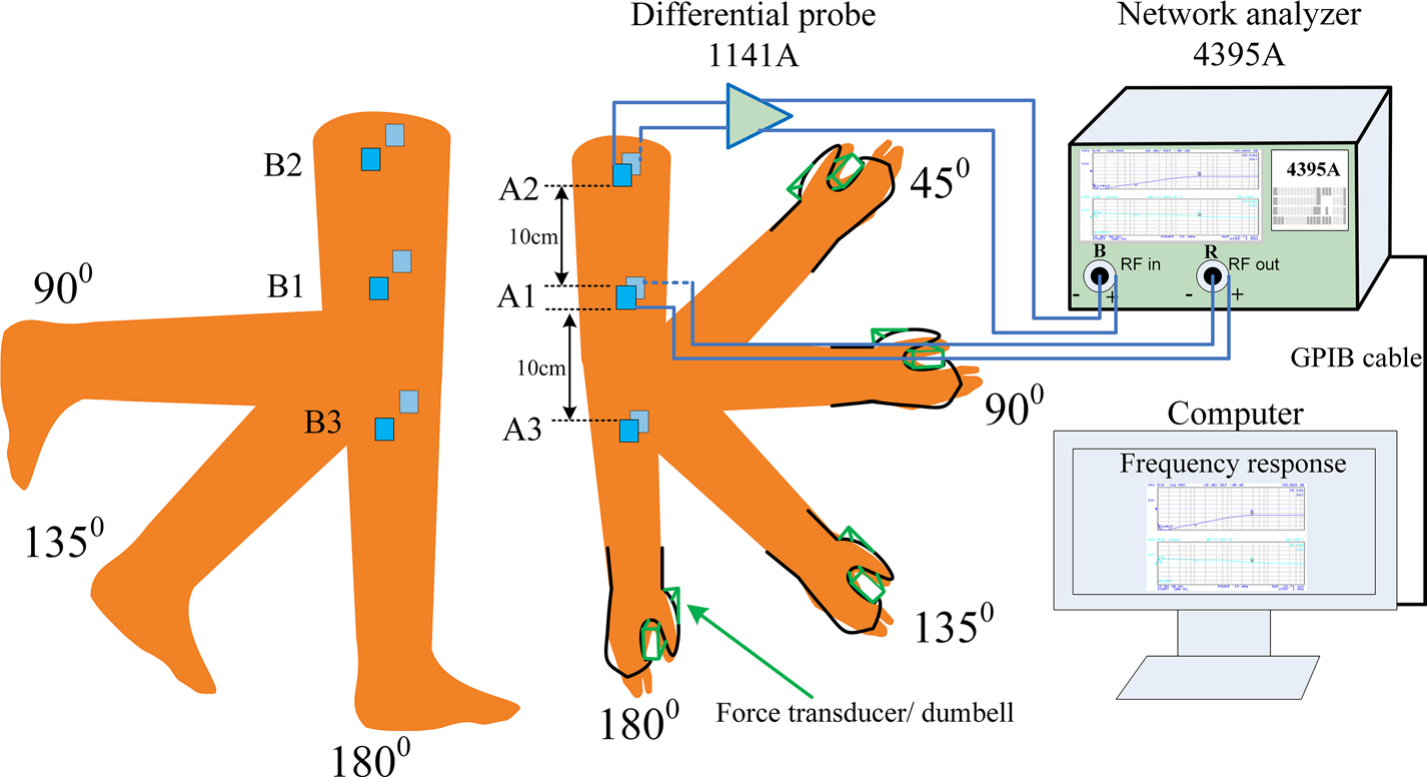
Experiment setup to investigate the effects of limb gestures on galvanic coupling IBC channel. For upper extremity channels (A1A2 and A1A3), the frequency response at each joint angle is measured with empty-handed, gripping force and loading. For lower extremity channels (B1B2 and B1B3), it is measured with the change of knee joint angles

**Fig. 2:**
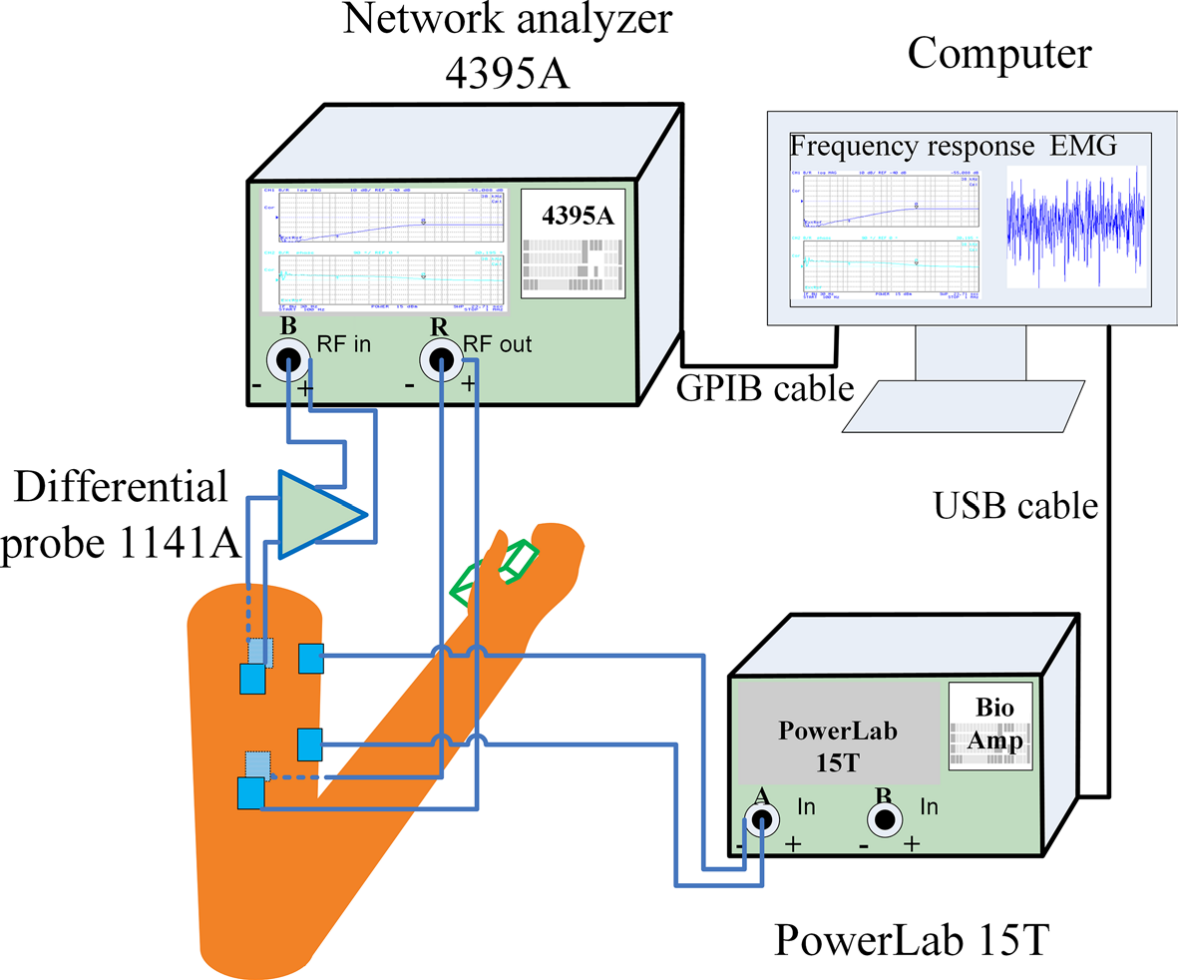
Experiment setup to investigate the effects of muscle fatigue on galvanic coupling IBC channel. With hand loading 2.5 kg dumbbell and elbow joint flexing to 45°, the EMG of biceps and frequency response of A1A2 are measured

Two experiment setups, shown in Figs. 1 and 2, were implemented for the purpose of studying the effects on galvanic coupling IBC channel from different limb gestures and muscle fatigue. For both experiments, the frequency response, which includes gain ($|H(f)|^2$) in dB and phase ($\theta \, (f)$) in degree, in sub-MHz frequency band was measured by a network analyzer (Agilent, 4395A). The sub-MHz frequency band is utilized as it can make the electric field confined within the human body [5], similar settings were adopted in other reports [7, 16, 24]. During measurements, the chirp signal from network analyzer was applied on the human limb by a pair of skin-attached stimulating electrodes (Shenzhen Jurongda Science and Technology Ltd., Carbon 20 × 20 mm), and detected by another pair of electrodes via a differential probe (Agilent, 1141A) that was used for breaking the ground loop of measurement equipment. For satisfactory received signal level, the vertical distance of the two pair of electrodes (channel length) was set to 10 cm. For the first experiment, as shown in Fig. 1, the measurements were performed on the upper and lower extremity channels including upper arm channel (A1A2), channel through elbow joint (A1A3), thigh channel (B1B2) and channel through knee joint (B1B3). For the upper extremity channels, different elbow joint angles (i.e. 180, 135, 90 and 45°) and hand conditions (i.e. empty-handed, loading with dumbbell and gripping a force transducer) were considered. For the lower extremity, different knee joint angles (i.e. 180, 135 and 90°) were evaluated. For the second experiment, as depicted in Fig. 2, wherein the measurements of frequency response and electromyography (EMG) of biceps [25, 26] were carried out on the upper arm. And the EMG was recorded by a bio-amplifier (NI instrument, PowerLab 15T) for further analysis.

**Fig. 3:**
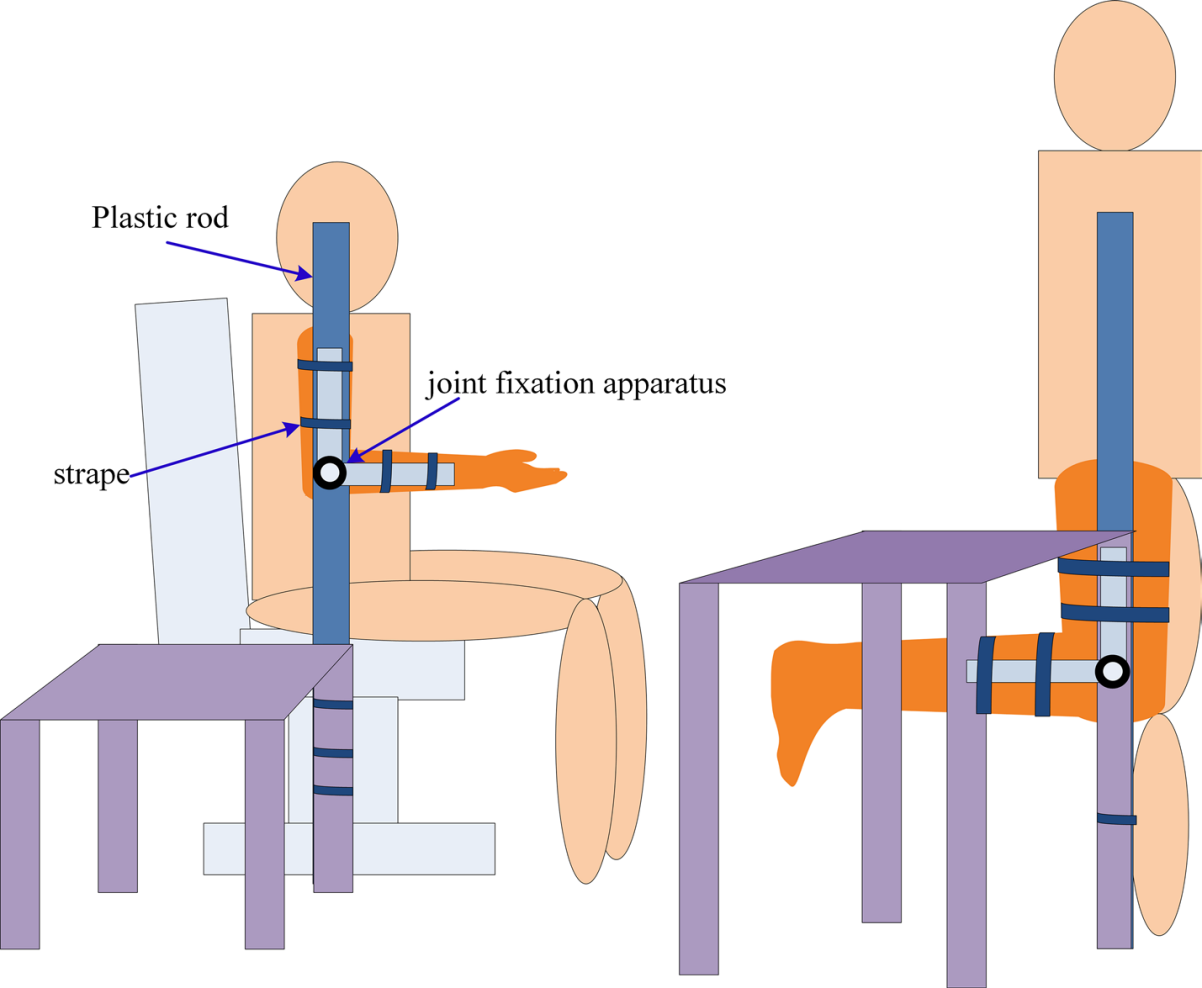
Experiment setup to avoid the movement of upper arm or thigh. For channels A1A2 and A1A3, the setup is adapted to avoid the movement of upper arm, while for B1B2 and B1B3, the movement of thigh is avoided

To prevent the movement of upper arm or thigh and at the same time maintain stable joint angle, a plastic rod (length: 1.2 m, diameter: 19 mm) sticking with a joint fixation apparatus (Hengshui Jingyuan Medical Apparatus and Instruments Ltd., Joint type medical external fixation support) was fabricated and shown in Fig. 3. The rod was stucken to the heavy desk by glue and meanwhile fasten by strape. Then the upper part of apparatus was vertically secured to the rod by glue and strape, the lower part was left for angle adjusting. Once the angle achieved, the rotation axis of apparatus was fasten by screws to avoid changing. During the measurement on upper extremity channels, the subject was ask to sit in chair with thigh horizontal, right upper arm leant on the rod and downward vertically against the rod. By adjusting the height of chair, the elbow joint was visually aligned with the rotation axis of apparatus. Then the upper arm was securely fastened to the rod, and the lower arm was fasten to lower part of apparatus via strape. Consequently, the upper arm could not swing or move, meanwhile the joint angle was sustained. Similarly, for the lower extremity, the subject stand and the thigh was fastened to the rod.

### Experiment protocol

Four volunteers, ranging from 23 to 33 years of age participated in the study. All subjects were healthy and had no known neuromuscular or joint muscle disorders at the time of the study. All subjects were gave informed consent the procedures approved by the Institutional Review Board of Medical Center Institutional, Shenzhen Polytechnic. The measurement parameters are summarized in Table 1. The anthropometrical characteristics of the subjects including the body mass index (BMI) are presented in Table 2. After the experiment setup, the subject performed ten trails of joint contraction and extension with joint angle changes from approximately 180–90 or 45°. With a 1 min rest, the subject got into the first experiment session: limb gesture effect experiment.

**Table 1:** The measurement parameters

Parameters	Value
Test subjects	Two males and two females
Signal transmission power	0 dBm
Frequency range	1 kHz–1 MHz
Electrodes	Stimulating electrodes (carbon)
Transverse distance between electrodes	Circular symmetry: 6–12 cm (upper limbs), 11–20 cm (lower limbs)
Transmission distance	10 cm

**Table 2:** The anthropometrical characteristics of subjects

Subjects	Age	BMI	Upper arm length (cm)	Upper arm diameter (cm)	Thigh length (cm)	Thigh diameter (cm)
S1^a^	33	18.1	20	6	35	11
S2	23	20.0	25	8	40	14
S3	26	23.2	25	9	41	16
S4^a^	25	26.3	22	12	35	20

^a^Represents female

#### Limb gesture effect experiment

The experiment setup is presented in Fig. 1, and the subject was instructed not to move. The frequency response of channel A1A2 with joint angle 180° was measured by the calibrated network analyzer with empty-handed, loading with 0.5 and 2.5 kg mass (dumbbell), gripping force of 0.5 and 2.5 kg. With each case, the subject held the gesture for 10 s to make sure the channel frequency response was steady for capture. So is the case with 135, 90 and 45°. Similarly, the experiments were performed on channel A1A3.

Finishing the measurement on upper extremity, the setup was changed to the lower extremity. Due to the limited motion of range in leg, three knee joint angles (180, 90 and 15°) are considered. After the limb gesture measurement, the subject got into the second experiment session: muscle fatigue effect experiment.

#### Muscle fatigue effect experiment

The experiment setup is shown in Fig. 2. From our empirical experiment, the human limb feels fatigue in 1 min when the hand is loading a 2.5 kg dumbbell. Consequently, the EMG of upper arm was recorded for 1 minute via the bio-amplifier with hand loading 2.5 kg dumbbell and elbow joint maintaining at 45°. Then the frequency response was captured at different recording time, for instance at 2, 30 and 58 s.

### Statistic method and signal processing

According to our empirical experiments (up to 23 subjects), the gain or phase follows approximately normal distribution among the subjects. Additionally, more than two groups of measurements were conducted for each effect. Consequently, one-way analysis of variance (ANOVA) with F test [27] was adopted to examine the significance of different gestures’ effects. The F statistic is calculated by dividing the mean square of the between-groups variance by the mean square of the within-groups variance. Then *p* value can be calculated from the F distribution based on the specific F statistic and the number of groups and observations.

To analyze the effects of channel variations on communication performance, the change of BER for some typical modulation schemes, such as quadrature phase shift keying (QPSK), 16-ary quadrature amplitude modulation (16QAM) and 16-ary frequency shift keying (16FSK) [28] was calculated. Assuming that the noise in IBC channel, which is mainly from the thermal noise of electronic devices and electrode-skin interface, is additive white Gaussian noise (AWGN) [24], the BER of the modulation methods is described as:

2 $$\begin{aligned} Pb_{QPSK}=Q \, (\sqrt{2\gamma _b}) \end{aligned}$$

3 $$\begin{aligned} Pb_{16QAM}=Q \, (\sqrt{0.8\gamma _b}) \end{aligned}$$

4 $$\begin{aligned} Pb_{16FSK}=15Q(\sqrt{4\gamma _b}) \end{aligned}$$

where *Pb* is the BER, *Q* is the Q-function [24]. $\gamma _b$ is signal to noise ratio per bit, which is proportional to channel gain. With a BER of $10^{-6}$, the required $\gamma _b$ is 10.5, 14.5 and 8.4 dB for QPSK, 16QAM and 16FSK, respectively. With the change of channel gain, the $\gamma _b$ will change, and thus BER will vary (e.g. channel gain attenuates 3 dB, $\gamma _b$ decreases 3 dB, BER increases from $Q \, (\sqrt{2\gamma _b})$ to $Q \, (\sqrt{2\times 10^{\frac{-3}{10}}\gamma _b})$ for QPSK).

## Results and discussions

### Results

The frequency response of galvanic coupling IBC channel affected by joint angle and muscle fatigue is depicted in this section. For the sake of repeatability, the measurements were carried out over 3 days, and the variations are calculated. To demonstrate the inter-subjects difference, the results at two angles (i.e. at 180 and 90°) from angle effect for different subjects are displayed. For sake of comparison among different effects, the mean of gain and phase over 3 days’ measurements influenced by joint angle, hand gripping force and loading are displayed in a table. The variations of BER performance due to extension of joint angle among the subjects are presented.

#### Effects of joint angle

**Fig. 4:**
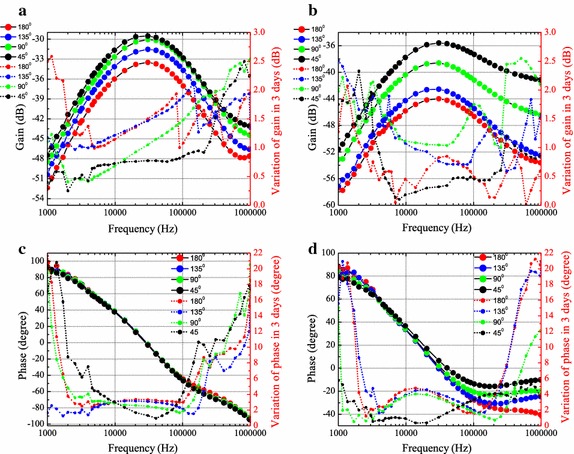
Gain and phase on upper extremity channels (A1A2 and A1A3) with various elbow joint angles. The* solid curves* with* symbols* refer to the* y-axis* on the* left*, depict the gain (**a** for A1A2,** b** for A1A3) and phase (**c** for A1A2,** d** for A1A3). The* dotted curves* with* symbols* refer to the* y-axis* on the* right*, represent the average of standard deviation from the measurements over 3 days

**Fig. 5:**
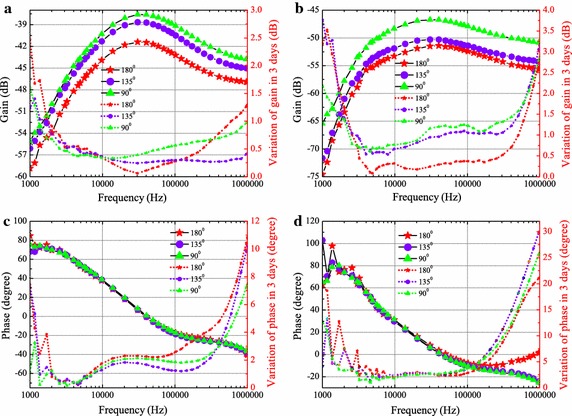
Gain and phase on lower extremity channels (B1B2 and B1B3) with various knee joint angles. The* solid curves* with* symbols* refer to the* y-axis* on the* left*, depict the gain (**a** for B1B2,** b** for B1B3) and phase (**c** for B1B2,** d** for B1B3). The* dotted curves* with* symbols* refer to the* y-axis* on the* right*, represent the average of standard deviation from the measurements over 3 days

The gain and phase on upper and lower extremity channels affected by joint angle (for subject 1) are shown in Figs. 4 and 5, respectively. The results reveal that there is a strong dependence between channel gain and joint angle, and generally the gain increases as the joint angle decreases. A bandpass profile with passband from 20 to 100 kHz is observed and peak is found at frequency around 20 kHz. Compared to Fig. 4a, more obvious gain increase is observed in Fig. 4b due to the decrease of angle, and more significant gain increase occurs at small joint angle positions (i.e. from 135 to 90° and from 90 to 45°). Similarly, in lower extremity channels, the gain is significantly increased at small angle position (from 135 to 90°) for Fig. 5b. It is noticed that the phase is less influenced by the joint angle. As shown in Figs. 4c and 5c, the phase curves is almost indistinguishable for various joint angles. For channels through the joint, the phase (illustrated in Figs. 4d, 5d) is just distinguishable in the relatively high frequency (i.e. higher than 200 kHz).

#### Variation on different days

The standard deviation (square root of the variance) over 3 days for subject 1 are included in Figs. 4 and 5. From the 3 days’ measurement results, the standard deviation of gain is lower than 3 dB, phase variation is lower than 22° for upper extremity channels. For lower extremity channels, the gain variation is lower than 4 dB, phase variation is lower than 30°. More noticeable variation is found in the low frequency (i.e. lower than 4 kHz) and high frequency regions (i.e. higher than 200 kHz). This could be explained by the fact that, the low frequency components are more prone to the environmental interference (i.e. noise, voice), while the high frequency components are affected by some parasitic capacitance (i.e. electrode-skin parasitic capacitance).

#### Different channels

Comparing Fig. 4a and b, with the same transmission distance 10 cm, the gain is lower in Fig. 4b, which means the appearance of elbow joint in IBC channel leads to an additional channel attenuation around 5 dB (at 45°) to even 10 dB (at 180°). For the lower extremity case, more than 3 dB is suffered from the the knee joint. Similar results are reported in [14]. This is mainly due to the large area of bone and seldom muscle in joint, which hinders the electric field penetration and electric current transfer.

With the same distance, for the channels without joint (A1A2 and B1B2) or with joint (A1A3 and B1B3), the lower extremity channel suffers from higher attenuation.

#### Different subjects

**Fig. 6:**
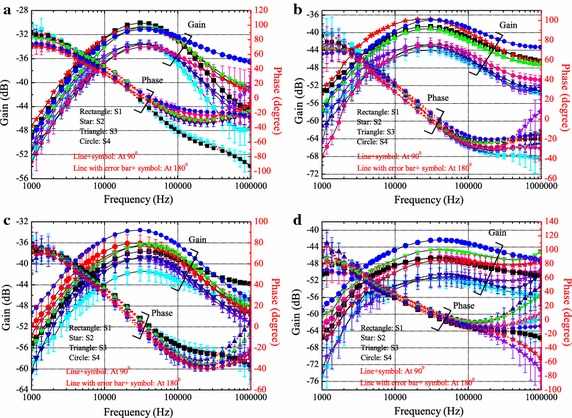
Gain and phase at two joint angles for the four subjects. The values at 90° are depicted by* lines* with* symbols*.* Lines* with* error bars* (variance over 3 days measurement) and symbols represent the values at 180°. The values from channel A1A2, A1A3, B1B2 and B1B3 are displayed in sub-figure **a**, **b**, **c** and **d**, respectively.* Lines* with* symbol rectangular*,* star*,* triangle* and* circle* is for S1, S2, S3 and S4, respectively

To demonstrate the inter-subjects difference, the gain and phase at two joint angles (90 and 180°) from the four subjects are shown in Fig. 6. With smaller joint angle, the gain is higher. The gains for different subjects are showed similar trends with variations lower than 5 dB when frequency lower than 200 kHz, larger variations are generally in the higher frequency regions (around 1 MHz). Higher gain is obtained for subject four (S4). This could be due to the larger diameter of limbs, similar results have also been reported by other authors [16].

#### Effects of loading, gripping force and muscle fatigue

**Fig. 7:**
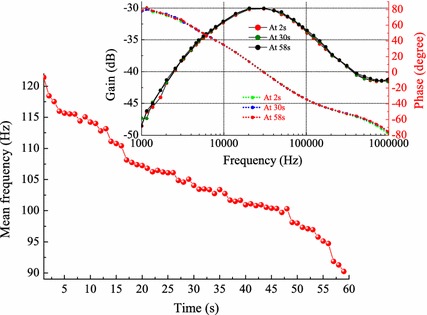
Muscle fatigue effect on IBC channel. In the subfigure with double* y-axis*, the gain is depicted by* solid curves* with* symbols*, while phase is depicted by* dotted curves*

The influence of muscle fatigue is shown in Fig. 7. The MNF of the EMG decreases from 120 to 90 Hz, which indicates the muscle is in the process of fatigue. While the gain and phase captured at different time is almost indistinguishable, which suggests that muscle fatigue has negligible effect on the IBC channel.

For hand loading and gripping force, their influence is small and the value will be shown in next section.

#### Comparison of different gestures

The effects of the three gestures on upper extremity channels from the four subjects are shown in Table 3. For joint angle effect, by using the baseline value with elbow joint angle 180°, deviation in gain and phase at 20 kHz for other joint angles (at 135, 90 and 45°) are calculated. For gripping force and loading (0.5 and 2.5 kg), baseline value of empty-handed is referred. Note that these values are the average of the three days’ measurements.

**Table 3:** Standard deviations and *p* values on gain and phase at 20 kHz for upper extremity IBC channels from different effects

Effect on subjects		Joint angle effect			Gripping force effect		Loading effect	
		At 135°	At 90°	At 45°	0.5 kg	2.5 kg	0.5 kg	2.5 kg
*Upper arm channel (A1A2)*								
S1	Gain (dB)	1.27	3.11	3.75	0.17	0.18	0.11	0.21
	Phase (degree)	1.42	2.85	2.16	2.65	2.41	0.55	0.97
S2	Gain (dB)	1.97	3.44	4.09	0.11	0.11	0.08	0.15
	Phase (degree)	1.15	1.97	3.21	0.82	0.79	0.30	0.69
S3	Gain (dB)	1.51	3.79	4.49	0.15	0.17	0.49	0.30
	Phase (degree)	1.25	2.14	3.24	0.25	0.28	0.64	0.97
S4	Gain (dB)	2.00	3.68	4.96	0.33	0.77	0.10	0.63
	Phase (degree)	2.21	2.91	2.10	0.83	0.68	0.41	0.54
*p* value	On gain	0.014			0.826		0.793	
	On phase	0.946			0.991		0.992	
*Channel through the joint (A1A3)*								
S1	Gain (dB)	3.89	6.24	9.56	0.35	0.38	0.76	0.53
	Phase (degree)	4.62	3.38	6.94	2.29	2.65	1.93	1.81
S2	Gain (dB)	3.20	7.46	11.70	0.47	0.38	0.33	0.43
	Phase (degree)	2.11	4.97	9.22	1.83	2.11	1.25	2.20
S3	Gain (dB)	1.09	4.36	8.11	0.19	0.36	0.26	0.13
	Phase (degree)	1.81	3.41	7.09	0.90	1.13	1.92	1.35
S4	Gain (dB)	1.34	5.68	10.51	0.26	0.24	0.30	0.68
	Phase (degree)	3.25	6.48	11.35	3.65	3.66	0.90	1.05
*p* value	On gain	0.001			0.904		0.987	
	On phase	0.899			0.836		0.913	

The *p* values of F test for the three effects are also included in Table 3. During the calculation, the gain and phase at 20 kHz serve as the data in observations. The number of groups is 4 (four joint angles), 12 (0, 0.5 and 2.5 kg at each angle) and 12 for angle effect, gripping force effect and loading effect, respectively. The respective number of observations is 16, 48 and 48. The *p* values smaller than 0.05 are considered statistically significant.

For joint angle effect, the trends from the four subjects are similar, that is the smaller the joint angle, the larger the gain variation. The change of joint angle causes great variations on channel gain, i.e., gain variation larger than 3.75 and 8.11 dB at 45° for A1A2 and A1A3, respectively. The joint angle factor has significant effect on gain (*p* < 0.014 for A1A2, *p* < 0.001 for A1A3). For gripping force and loading, their effects are small, i.e. gain variation smaller than 0.77 dB (*p* > 0.793), phase variation lower than 4° (*p* > 0.836), and can be ignored.

#### Variation of bit error rate

For human limb IBC channels, the worse channel condition (lowest gain) occurs when the joint extends to position 180°. While best channel condition is obtained when joint flexes, i.e. to position 45° for upper extremity channels, 90° for lower extremity channels. With joint’s flexion and extension, the channel suffers from unstable channel conditions, which will cause the communication performance (i.e. BER) to vary.

Assuming that BER is 10$^{-6}$ when channel is at best channel condition (at 45° for A1A2 and A1A3, at 90° for B1B2 and B1B3), BERs at worse channel conditions for the four subjects are displayed in Table 4. The results show that in subject 1 (S1), in worse case, BER of QPSK degrades to 1.0 × 10^−3^ and 5.7 × 10^−2^ for A1A2 and A1A3 respectively. And the degradation is less significant for lower extremity channels. It can be noticed that the flexion and extension of elbow joint causes a one ( 1.5 × 10^−5^–1.0 × 10^−6^) to five (1.0 × 10^−1^–1.0 × 10^−6^) orders of magnitude variation in BER of QPSK for upper extremity channels, while for lower extremity channels, the variation is over one (1.2 × 10^−5^–1.0 × 10^−6^) to three (9.3 × 10^−3^–1.0 × 10^−6^) orders of magnitude due to the change of knee joint angle.

**Table 4:** BER performance of modulation schemes with joint extending to different positions (for upper limb channels, BER is $10^{-6}$ with elbow joint at 45°, for lower limb channels, BER is $10^{-6}$ with knee joint at 90°)

Subjects	A1A2			B1B2	
	To 90°	To 135°	To 180°	To 135°	To 180°
*QPSK*					
S1	2.1 × $10^{-5}$	4.6 × $10^{-4}$	1.0 × $10^{-3}$	1.2 × $10^{-5}$	1.0 × $10^{-4}$
S2	7.9 × $10^{-5}$	7.1 × $10^{-4}$	1.5 × $10^{-3}$	4.7 × $10^{-5}$	1.3 × $10^{-4}$
S3	3.4 × $10^{-5}$	1.0 × $10^{-3}$	2.3 × $10^{-3}$	4.9 × $10^{-5}$	3.0 × $10^{-4}$
S4	8.3 × $10^{-5}$	9.6 × $10^{-4}$	3.7 × $10^{-3}$	1.9 × $10^{-5}$	1.6 × $10^{-4}$

Subjects	A1A3			B1B3	
	To 90°	To 135°	To 180°	To 135°	To 180°
S1	1.2 × $10^{-3}$	1.0 × $10^{-2}$	5.7 × $10^{-2}$	6.8 × $10^{-4}$	3.6 × $10^{-3}$
S2	5.2 × $10^{-4}$	2.2 × $10^{-2}$	1.0 × $10^{-1}$	3.0 × $10^{-4}$	1.3 × $10^{-3}$
S3	1.5 × $10^{-5}$	2.1 × $10^{-3}$	3.1 × $10^{-2}$	2.0 × $10^{-3}$	9.3 × $10^{-3}$
S4	2.4 × $10^{-5}$	6.8 × $10^{-3}$	7.9 × $10^{-2}$	4.7 × $10^{-4}$	1.4 × $10^{-3}$

Subjects	A1A2			B1B2	
	To 90°	To 135°	To 180°	To 135°	To 180°
*16QAM*					
S1	2.0 × $10^{-5}$	4.5 × $10^{-4}$	1.0 × $10^{-3}$	1.2 × $10^{-5}$	1.0 × $10^{-4}$
S2	7.7 × $10^{-5}$	7.0 × $10^{-4}$	1.5 × $10^{-3}$	4.6 × $10^{-5}$	1.3 × $10^{-4}$
S3	3.3 × $10^{-5}$	1.1 × $10^{-3}$	2.3 × $10^{-3}$	4.7 × $10^{-5}$	3.0 × $10^{-4}$
S4	8.1 × $10^{-5}$	9.4 × $10^{-4}$	3.6 × $10^{-3}$	1.8 × $10^{-5}$	1.6 × $10^{-4}$

Subjects	A1A3			B1B3	
	To 90°	To 135°	To 180°	To 135°	To 180°
S1	1.2 × $10^{-3}$	1.0 × $10^{-2}$	5.7 × $10^{-2}$	6.7 × $10^{-4}$	3.6 × $10^{-3}$
S2	5.1 × $10^{-4}$	2.2 × $10^{-2}$	1.1 × $10^{-1}$	3.0 × $10^{-4}$	1.3 × $10^{-3}$
S3	1.4 × $10^{-5}$	2.0 × $10^{-3}$	3.1 × $10^{-2}$	1.9 × $10^{-3}$	9.2 × $10^{-3}$
S4	2.4 × $10^{-5}$	6.8 × $10^{-3}$	7.8 × $10^{-2}$	4.6 × $10^{-4}$	1.4 × $10^{-3}$

Subjects	A1A2			B1B2	
	To 90°	To 135°	To 180°	To 135°	To 180°
*16FSK*					
S1	4.1 × $10^{-5}$	1.8 × $10^{-3}$	4.7 × $10^{-3}$	2.1 × $10^{-5}$	2.9 × $10^{-4}$
S2	2.1 × $10^{-4}$	3.0 × $10^{-3}$	7.6 × $10^{-3}$	1.1 × $10^{-4}$	4.1 × $10^{-4}$
S3	7.4 × $10^{-5}$	5.0 × $10^{-3}$	1.3 × $10^{-2}$	1.1 × $10^{-4}$	1.1 × $10^{-3}$
S4	2.2 × $10^{-4}$	4.3 × $10^{-3}$	2.2 × $10^{-2}$	3.5 × $10^{-5}$	4.8 × $10^{-4}$

Subjects	A1A3			B1B3	
	To 90°	To 135°	To 180°	To 135°	To 180°
S1	5.8 × $10^{-3}$	7.7 × $10^{-2}$	6.1 × $10^{-1}$	2.8 × $10^{-3}$	2.2 × $10^{-2}$
S2	2.0 × $10^{-3}$	1.9 × $10^{-1}$	1.28	1.1 × $10^{-3}$	6.4 × $10^{-3}$
S3	2.6 × $10^{-5}$	1.1 × $10^{-2}$	2.9 × $10^{-1}$	1.0 × $10^{-2}$	6.7 × $10^{-2}$
S4	4.9 × $10^{-5}$	4.7 × $10^{-2}$	8.8 × $10^{-1}$	1.8 × $10^{-3}$	6.8 × $10^{-3}$

Among the three modulation schemes, 16FSK is most susceptible to the influence of joint angle (largest BER variation). QPSK and 16QAM obtains almost the same BER performance variation.

### Discussion

#### Different effects

Now we have a brief summary, the flexion of elbow joint angle significantly affects channel gain (*p* < 0.014). The larger the joint extends, the higher the channel attenuates. The channel remains almost the same for gestures of empty-handed, loading and gripping force. These phenomenons can be explained by the fact that in galvanic coupling IBC channel, the majority of electric current is conveyed by muscle tissue (larger than 90 % for frequency lower than 1 MHz) [6]. When the elbow joint flexes (angle decrease), the muscle performs the concentric contraction, in which the length of muscle is shorten significantly [29], and thus results in lower channel attenuation (shorter channel length). While for hand loading or gripping force, the muscle undergoes the isometric contraction, wherein the reduction of muscle length is negligible [29, 30], and thus it leads to small changes of channel attenuation.

The findings suggest that in case of body movement without changing joint angle, the gain will be the same for different gestures. That explains why the gain of arm channels is approximately the same for the three positions of sitting, standing and walking in [16].

#### Practical hints for system design

For different gestures or moving conditions, the suitable frequency range for data transmission remains the same, that is from 20 to 100 kHz. Among the three modulation methods, QPSK is suitable for data transmission, since its high power efficiency [28] and relatively low BER variation. In worst case, the BER in upper limb channels will variate in five orders of magnitude for patient’s daily activity with joint angle changes. Therefore, to achieve the low power transmission and stable communication performance, the adaptive power control is required.

## Conclusion

The experiments to evaluate the effect of human limb gestures on galvanic coupling IBC channel have been carried out in this work. The hand loading, gripping force, as well as muscle fatigue has negligible effect on human limb IBC channel. While the joint has significant effect on channel gain. The channel suffers from highest attenuation when the joint extends to 180°, and obtains best channel condition when joint flexes to 45° for the upper extremity, 90° for the lower extremity. It can be concluded that the gain variation is mainly due to the change of joint angle, which suggests that the change of gain during body movement is mainly from the change of joint angle. In case of body movement without changing joint angle, the gain will be the same for different gestures. For various gestures, the suitable frequency band for data transmission is from 20 kHz to around 100 kHz. The gain variation causes significant BER variation in modulation schemes, which suggests that to enable power saving and stable communication performance, adaptive power control is recommended.
